# Patient preferences and health state utilities associated with the treatment process of antiretroviral therapy for people living with HIV

**DOI:** 10.1007/s11136-022-03290-0

**Published:** 2022-12-13

**Authors:** Louis S. Matza, Timothy A. Howell, Vasiliki Chounta, Nicolas van de Velde

**Affiliations:** 1 Patient-Centered Research, Evidera, Bethesda, MD USA; 2 Global Health Outcomes, ViiV Healthcare, London, England UK; 3 Formerly With Global Health Outcomes, ViiV Healthcare, London, England UK

**Keywords:** Health state utility, Treatment process utility, Utility, Route of administration, HIV, Antiretroviral therapy, Long-acting injectable

## Abstract

**Purpose:**

People living with HIV (PLHIV) have reported challenges associated with daily oral antiretroviral therapy (ART), including missed doses, negative psychological impact, and difficulty remaining discreet while at home or traveling. Recently approved long-acting injectable (LAI) ART may help eliminate these concerns. The purpose of this study was to examine patient preferences and estimate health state utilities associated with oral and LAI treatment for ART.

**Methods:**

Four health state vignettes were developed based on published literature, clinician interviews, and a pilot study. All vignettes included the same description of HIV, but differed in treatment regimens: (A) single daily oral tablet, (B) two daily oral tablets, (C) injections once monthly, and (D) injections every two months. PLHIV in the UK reported their preferences and valued the health states in time trade-off utility interviews.

**Results:**

The sample included 201 PLHIV (83.1% male; mean age = 44.9y). The health states frequently selected as most preferable were D (*n* = 119; 59.2%) and A (*n* = 75; 37.3%). Utility differences among health states were relatively small, which is typical for treatment process utilities (mean utilities: A, 0.908; B, 0.905; C, 0.900; D, 0.910). Statistically significant differences in utility were found for one vs. two tablets and injections every month vs. every two months (*p* < 0.001). Participants’ quotations highlight the wide range of reasons for treatment process preferences.

**Conclusions:**

Current results indicate that many PLHIV would prefer LAI ART. The reported utilities may be useful in economic modeling comparing oral vs. LAI ART.

**Supplementary Information:**

The online version contains supplementary material available at 10.1007/s11136-022-03290-0.

## Introduction

For people living with HIV (PLHIV), antiretroviral therapy (ART) can improve survival, suppress viral load to the point that it is undetectable, and eliminate the risk of passing HIV to others through sexual transmission [1–5]. Until recently, ART was available only in oral treatment formulations, often administered as one or two tablets taken once per day [6–9]. While these oral treatment regimens are more convenient than earlier ART formulations with more complex dosing schedules, patients have reported challenges associated with daily oral treatment.

For example, some PLHIV have reported unintentionally missing medication doses, which can affect treatment effectiveness [10–14]. PLHIV have also said oral medication is an unwanted daily reminder of HIV, while others have described inconvenience, annoyance, lack of freedom, and worry associated with the daily medication [12, 15, 16]. The stigma and discrimination associated with HIV presents another challenge [17–20]. Because of this stigma, some PLHIV avoid revealing their HIV status to other people, and daily oral medication can make it difficult to remain discreet at home and while traveling [12, 15, 16].

Recently approved long-acting injectable (LAI) ART provides an alternative treatment approach that may be appropriate for PLHIV who have these concerns [21, 22]. The combination of cabotegravir and rilpivirine, administered every four or eight weeks, has demonstrated efficacy and safety [23–25] with high levels of treatment satisfaction and acceptance [26]. Patients have reported a range of benefits of LAI dosing compared to daily oral treatment, including greater convenience, less worry about missing a dose, less opportunity for unwanted disclosure of HIV status, and a greater sense of psychological freedom without the daily reminder of HIV [15, 16, 27].

As LAI ARTs are considered for use in various countries, cost-utility analyses (CUA) will be needed to assess their value and inform decision-making about healthcare resource allocation. CUAs require utilities, which quantify the strength of preference for various health states [28]. The purpose of the current study was to examine preferences and estimate health state utilities associated with oral and LAI treatment processes for ART in a sample of PLHIV in the UK.

Research on “treatment process utilities” has shown that treatment characteristics like route of administration and dose frequency have an impact on preference and utility [29, 30]. It is often useful to include these utilities in CUAs to better represent the experience of people receiving treatment. In addition to utility estimates, the study results can provide insight into the preferences of PLHIV. These preferences are important to consider in clinical settings where they could have an impact on treatment adherence and treatment outcomes [31–33].

## Methods

### Overview of study design

Like most studies designed to estimate treatment process utilities [29, 34], this study was conducted using vignette-based methods. Generic preference-based measures such as the EQ-5D were designed to assess overall health status and are unlikely to be sensitive to differences in treatment process. In contrast, the vignette-based approach is well suited for isolating the impact of treatment process on utility.

Four health state vignettes were developed and refined based on published literature, clinician interviews, and a pilot study. All health states included the same description of a person living with HIV, but differed in the description of the treatment process. The four treatment processes were selected to represent two common oral ART regimens and two LAI regimens [10, 22, 24, 25, 35]. The health states were valued in a time trade-off (TTO) utility elicitation study with a sample of PLHIV in the UK. One-on-one TTO interviews were conducted by videoconference from November 2020 to January 2021. Because the four health states varied only in the treatment process, all resulting differences in preference and utility can be attributed entirely to these treatment process differences.

Informed consent was obtained prior to each interview, and the study protocol was approved by an institutional review board (Ethical and Independent Review Services; Study 20173–01).

### Health state development

A targeted literature review was conducted to inform the initial draft of the health states. This review focused on the experience of living with HIV [4, 10, 17, 18, 36], ART [10, 12, 36, 37], and the ART treatment process [12, 15, 16, 38–41]. Based on this literature, initial drafts of four health states were developed, along with a detailed background information document providing details on the four ART options. Then, interviews were conducted with four clinicians (three HIV specialists and one infectious disease specialist; two MD, one MBBS, one MB BCh; three from London, UK, and one from Omaha, Nebraska) to refine the health states and background information document. These clinicians reported treating between 10 and 200 PLHIV per month, including 95% taking ART and over 90% at undetectable viral loads. Clinicians averaged over 22 years of experience working with HIV.

These interviews were conducted using a semi-structured interview guide that included questions on the clinicians’ professional background, description of HIV with an undetectable viral load, and description of daily oral and LAI ART. Health states were developed in an iterative process. Clinicians were interviewed multiple times, and interviews continued until clinicians agreed that the health states and background information document accurately described typical patient experiences with ART treatment regimens.

All four health states began with the same description of a person living with HIV, treated with ART and having an “undetectable viral load.” After the description of HIV, each health state described one of four treatment processes. Health state A described a single tablet taken daily, and health state B described two tablets taken together daily. Both descriptions also stated that the tablets should be taken at the same time every day, and that the tablets need to be carried while traveling to ensure that treatment is not skipped on those days.

Health states C and D described injections administered monthly and once every two months, respectively. These descriptions stated that the injections must be administered at a clinic or hospital, must occur around the same day each month, are administered in the buttocks, and only take a few minutes. The health states also specified that if an individual is receiving injections as scheduled, there is no need for additional oral medication. Complete health state text is presented in the online supplementary material.

A background information document (included in supplementary material) was developed to inform respondents about potential advantages and disadvantages of oral and LAI routes of administration for ART. This background information page was developed to ensure that participants were aware of the potential implications of the two routes of administration before they were asked to report their health state preferences. To ensure that respondent preferences were based on the treatment process and not on assumptions of drug efficacy or adverse event profiles, the background information document stated that the treatments were equally effective with similar rates of side effects, but that “research has shown that some patients prefer the daily oral treatment, while others prefer the long-acting injectable treatment.” A series of bullet points then described some of the primary reasons that PLHIV have reported for liking/disliking the two routes of administration (three positive points and three negative points for each route of administration). Participants were instructed to consider these advantages and disadvantages of the two routes of administration during the health state ranking and utility elicitation. All treatment attributes in this background information document were selected based on published literature [12, 15, 16, 37, 38] and clinician input. The clinicians helped to refine this document so that it was consistent with their observations of typical patient experiences.

### Pilot study

A pilot study was conducted with 16 PLHIV (75.0% male; mean age = 39.5 years) in the UK. Participants completed a TTO valuation and provided feedback on the health states and procedures. Based on this feedback, health states were edited for clarity and ease of understanding. The participants often reported that the description of living with HIV was similar to their own experience. All participants said the health states and background information document were clear and easy to understand. Data from the pilot study were not included in the main valuation analysis sample.

### Participants

Participants were recruited from a patient database that was populated as follows: collaborating with patient support organizations and charities, digital marketing to targeted audiences (e.g., via Facebook, Twitter, Google), and patient referrals. Recruitment messages for the current study were sent via email to PLHIV in the database. If participants responded with interest, they were screened by phone for eligibility. To be eligible for this study, participants were required to be over 18, a UK resident, diagnosed with HIV, virologically suppressed (i.e., viral load under 50 copies per milliliter) as indicated by their most recent test, and currently treated with ART. Because interviews were conducted by videoconference, participants were required to have a desktop computer, laptop, or tablet with video capabilities, and have a physical mailing address where they could receive a packet of study materials. Respondents were required to provide proof of receiving ART for HIV in one of three ways: a photo of the medication or medication packaging, a prescription note containing their name and the medication name, or a letter from their hospital/clinic/doctor/nurse.

### Utility interview procedures and scoring

The four health states finalized in the pilot study were used to assess preference and elicit health state utilities in the larger utility elicitation. Six trained interviewers conducted one-on-one interviews, following a semi-structured interview guide. Each interviewer was observed by the principal investigator (PI) at least once (by joining the Microsoft Teams meeting) to ensure consistency in interview procedures.

First, participants were introduced to the differences between oral and LAI treatment process, using the background information document. The interviewer reviewed each bullet point in this document to ensure that the respondent understood these details. Then, the four health states were presented in random order and reviewed at the same level of detail. The participant was then given an opportunity to read the materials independently and ask questions about the health state content. After confirming that the participant understood the health states and had no further questions, participants were asked to rank the health states from most preferable to least preferable and explain their preferences. Direct quotes were recorded by the interviewer.

After completing the ranking, participants valued the health states in a TTO task with a 20-year time horizon. TTO methods have been described extensively in previous publications [28]. For each health state, participants were offered a series of choices between a life of 20 remaining years in the health state being rated or a shorter period of time in full health, followed by dead. Choices were presented in a way that alternated between longer and shorter periods of time in full health (e.g., 20 years, 0, 1, 19, 2, 18, 3…). Each health state received a utility value on a scale with the anchors of dead (0) to full health (1) based on the choice in which the respondent was indifferent between 20 years in the health state being evaluated and *x* years in full health. The resulting utility estimate (*u*) is calculated as *u* = *x*/*20*.

Different TTO procedures were planned for health states perceived to be worse than dead. However, no participants perceived a health state to be worse than dead, so these procedures were never implemented.

### Videoconference interview procedures

To maintain data quality and ensure the respondents fully understand the health state vignettes and TTO task, the optimal approach for vignette-based utility elicitation involves one-on-one interviews in which the interviewer and respondent can share the study materials. Because of the COVID-19 pandemic, however, interviews could not be conducted in person. Therefore, a three-step approach was used to simulate the experience of an in-person interview. First, a package was sent to the participant with paper copies of all materials that would be necessary for the interview, including the health states, the background information document, and questionnaires. Participants were instructed not to review any of the materials prior to the interview.

Second, the one-on-one interviews were conducted by videoconference via Microsoft Teams. At the beginning of the interview, participants were instructed to open the package that had been sent to them. They were also asked to work at a table or desk large enough for the four health states to be spread out and placed in order of preference. With the videoconferencing software, the interviewer and participant could see each other and point to parts of the health states and background information document as if they were working together in the same room. The TTO choices (e.g., 20 years in full health vs. a shorter amount of time in the health state being rated) were presented in a PowerPoint slide deck with the screen sharing feature of Microsoft Teams.

Third, the PI and/or project manager (PM) were available for questions during every interview. For example, if an interviewer was unsure about how to answer a participant’s question or how to clarify an aspect of the TTO task, the interviewer would send a text message to the PI or PM for assistance. If a brief response would suffice, the PI or PM could respond by text message. If a more thorough response was necessary, the PI or PM could join the Microsoft Teams meeting to help clarify the issue. With this approach to supervision, it was possible to simulate the experience of collecting data as a team in a single location, thus maximizing data quality and consistency across interviewers.

### Patient-reported questionnaires

After the utility elicitation, participants completed two questionnaires. First, they completed a demographic and clinical form. Second, they reported health-related quality of life on the SF-12 [42]. The SF-12 has been found to be a valid and reliable measure in PLHIV [43–46].

### Statistical analysis procedures

Statistical analyses were conducted with SAS version 9.4. Descriptive statistics were used to summarize demographic data, SF-12 physical/mental component summary scores, health state preferences, and utilities (frequencies and percentages for categorical variables; means and standard deviations for continuous variables). Paired t-tests were conducted to examine differences between utility means (e.g., utility of health state A vs. utility of health state B), and independent t-tests were used to test for subgroup differences in utilities by age [median split], gender, and employment status [employed vs. not employed]). Post hoc descriptive analyses were conducted to provide utilities for subgroups of patients categorized based on preference for oral or LAI treatment.

## Results

### Sample characteristics

A total of 268 potential participants were eligible based on screening. Of those, 226 were scheduled, and 205 attended interviews. Four of these participants who were eligible at screening became ineligible by their interview date because of test results indicating that they were no longer virologically suppressed. Therefore, the analysis includes data from 201 interviews. See Table 1 for demographics.

**Table 1:** Sample Characteristics

Characteristics	Descriptive Statistics (*N* = 201)
Age (mean, median [SD])	44.9, 45.0 (11.1)
Gender (*n*%)	
Male	167 (83.1%)
Female	32 (15.9%)
Transgender (Male to Female)	1 (0.5%)
Nonbinary	1 (0.5%)
Ethnicity (*n*%)	
White	157 (78.1%)
African, Caribbean, or Black	20 (10.0%)
Asian	3 (1.5%)
Mixed race^a^	15 (7.5%)
Other^b^	6 (3.0%)
Marital Status (*n*%)	
Single	130 (64.7%)
Married/Cohabitating/Living with partner	64 (31.8%)
Other	7 (3.5%)
Sexual Orientation (*n*%)	
Heterosexual/Straight	35 (17.4%)
Homosexual/Gay/Lesbian	154 (76.6%)
Bisexual	8 (4.0%)
Asexual	1 (0.5%)
Other^c^	3 (1.5%)
Employment Status (*n*%)	
Full-time work	110 (54.7%)
Part-time work	30 (14.9%)
Other^d^	61 (30.3%)
Education Level (*n*%)	
University degree or higher	111 (55.2%)
No university degree	90 (44.8%)
Geographical Location (*n*%)	
England	188 (93.5%)
Scotland	10 (5.0%)
Wales	2 (1.0%)
Northern Ireland	1 (0.5%)

^a^Mixed race includes 'Anglo-Indian' (*n* = 1), 'Arab and White' (*n* = 1), 'British and Puerto Rican' (*n* = 1), 'English and Peruvian' (*n* = 1), 'Italian and Black Caribbean' (*n* = 1), 'Latino, Black, White, Indigenous' (*n* = 1), 'Latino and Asian' (*n* = 1), 'Multiracial' (*n* = 1), 'Nigerian and British' (*n* = 1), 'White British and Black African' (*n* = 1), 'White and Asian' (*n* = 2), 'White and Black Caribbean' (*n* = 1), 'White and Indian' (*n* = 1), and 'White and Black African' (*n* = 1)

^b^Other ethnicity includes 'Chinese' (*n* = 1), 'Hispanic' (*n* = 1), 'Latino' (*n* = 2), 'Mediterranean' (*n* = 1), and 'South Asian' (*n* = 1)

^c^Other sexual orientation includes 'Don't agree with labeling, sexuality is a spectrum' (*n* = 1) and 'Queer' (*n* = 2)

^d^Other employment status includes carer (*n* = 2), disabled (*n* = 17), homemaker (*n* = 1), retired (*n* = 15), self-employed (*n* = 5), student (*n* = 3), unemployed (*n* = 17), and volunteer (*n* = 1)

*SD* standard deviation

Participants reported being diagnosed with HIV an average of 14.1 (SD = 8.7) years prior to their interview and beginning ART an average of 11.3 (SD = 6.8) years prior. Almost all participants were currently on a regimen of one pill per day (*n* = 92; 45.8%) or multiple pills taken at the same time daily (*n* = 99; 49.3%). Most participants (*n* = 187; 93.0%) reported missing their medication fewer than one day per week. Only one participant (0.5%) reported having used LAI ART. The most commonly reported comorbid health conditions were anxiety (*n* = 78; 38.8%), depression (*n* = 67; 33.3%), hypertension (*n* = 25; 12.4%), and arthritis (*n* = 19; 9.5%). Mean (SD) SF-12 Mental and Physical Component Scores were 50.38 (12.14) and 42.74 (10.25), respectively. These scores are somewhat lower (i.e., indicating worse health) than those found in other studies reporting SF-12 values of PLHIV [44], but this study occurred during the COVID-19 pandemic, which could have affected these scores.

### Health state rankings and preferences

Participants ranked the health states from most preferable to least preferable (Table 2). Almost all participants said the most preferable health state was either D representing injections once every two months (*n* = 119; 59.2%) or A representing a single daily tablet (*n* = 75; 37.3%). Preference orders (listed in order from most to least preferable) reported by at least 5% of the sample included DCAB (*n* = 70; 34.8%), ABDC (*n* = 58; 28.9%), DABC (*n* = 31; 15.4%), DACB (*n* = 14; 7.0%), and ADBC (*n* = 10; 5.0%).

**Table 2:** Health state rankings^a^ (*N* = 201)

	Frequency of rankings (n%)			
Health States	1 = Most preferred	2	3	4 = Least preferred
A. One daily tablet	75 (37.3%)	46 (22.9%)	74 (36.8%)	6 (3.0%)
B. Two daily tablets	3 (1.5%)	60 (29.9%)	45 (22.4%)	93 (46.3%)
C. Injections every month	4 (2.0%)	73 (36.3%)	23 (11.4%)	101 (50.2%)
D. Injections once every two months	119 (59.2%)	22 (10.9%)	59 (29.4%)	1 (0.5%)

^a^Prior to the time trade-off utility elicitation, participants were asked to rank the health states in the order of most preferable to least preferable. Rankings are summarized in this table, with lower numbers indicating more preferred health states

After providing their rankings, participants explained their preferences. Common reasons for preferring daily oral treatment included the ease of oral administration, convenience of daily tablets, fear of needles, and the fact that oral medication is taken at home without requiring additional medical appointments. Common reasons for preferring LAI administration were the convenience of less frequent dosing, no risk of missing a daily dose, elimination of the daily reminder of HIV, and the convenience of traveling without bringing medication. Quotations from participants are presented in Table 3.

**Table 3:** Selected Quotations from Participants Explaining Their Preferences Among the Four Health States

Two Groups of Participants	Selected Quotations
Participants who preferred daily oral treatment over injections every two months	“It is the inconvenience and pressure of appointment time and time off [required with the injection]. In my line of work that isn’t feasible.” “It’s a hassle to visit the clinic [for the injection]. Now I only visit the clinic once each year. The tablet gives me more flexibility than the injection.” “Tablets don’t interfere with my current lifestyle, and I don’t have trouble remembering them.” “With the tablets you don’t have to go to as many clinic appointments.” “There is pain with the injections. Getting into the clinic is very inconvenient and you have to go in often.” “The injections are unfamiliar. I have some fear of the unknown. I’ve never even heard of that [i.e., the injections]. I’d want to hear from peers that try it first.” “I take my tablets daily with my vitamins and it is easy.” “I’m traumatized by injections. That’s how scared I am. I just fear injections.” “Main objection to the injections is the time to get to the clinic. It would take 2 hours out of my day. I see the appeal of the injections, but the period in between would need to be longer for me.” “I hate needles. That’s a major issue for me. Especially stuff going in. Blood draws are bad enough, but injections are hell.” “Also, my clinic is in [location] and it is quite far away, so it isn’t convenient to go in every 2 months [for injections].” “I wouldn’t want to have to rely on getting to the clinic because I do international traveling.” “Arranging time off work to get the injections would be a pain.” “It is simply more convenient for me to take daily tablets than worry about making hospital appointments. Tablets are something I know I can do and fit into my routine.”
Participants who preferred injections every two months over daily oral treatment	“With daily [oral] treatment, there’s always a risk of missing a dose.” “The injectables are less to think about. I used to struggle with remembering to take my tablets.” “[I] prefer not to have medication every day.” “Sometimes the tablet is a daily reminder that you’re HIV positive. The injections would be just like a regular check-up. People would not know the appointment was for HIV, like if you don’t want to disclose at work.” “Seems a lot more convenient to have the injection less frequently than the tablets.” “I am bad at taking my meds at the same time every day.” “Injectables are easy to schedule.” “The clinic appointments wouldn’t be as intrusive as daily oral medication.” “Injections would be a lot easier. I wouldn’t have to worry about tablets. I hate taking tablets. I’m so anti-tablet. They remind me every day that I have HIV.” “I’m very used to injections and have no problem with them whatsoever.” “I’ve been on tablets for 15 years and sometimes I forget them which causes anxiety. And the daily reminder isn’t nice.” “I do a lot of traveling; I would love not having to take meds with me. There are a lot of countries that ban the medications.” “I like the idea of life without tablets.” “I don’t mind injections especially if it meant I wouldn’t have to take tablets anymore.” “I am less likely to forget. When you invite people over and they snoop, they won’t find anything.” “I’m a nightmare taking tablets. It’s really hard for me, plus I forget.” “I like the idea of the injection. Sometimes I forget my medication when I stay over at my dad’s.”

### Health state utilities

**Fig. 1:**
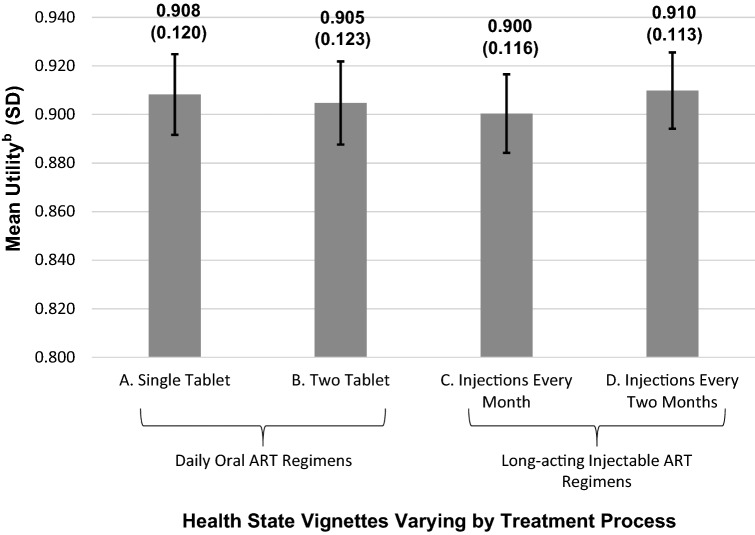
Mean Health State Utilities^a^ (*N* = 201). **a** TTO health state utilities are on a scale anchored with 0 representing dead and 1 representing full health. **b** The error bars represent the 95% confidence intervals for health state utilities: A, 0.892—0.925; B, 0.888—0.922; C, 0.884—0.917; D, 0.894—0.926

Health state D (injections every two months) had the highest mean utility score at 0.910, followed by A (single tablet daily) at 0.908, B (two tablets daily) at 0.905, and C (injections every month) at 0.900 (Fig. 1). Of the 201 respondents, 128 (63.7%) had the same utility score for all four health states, while 73 (36.3%) had differences in utility scores. Significant differences in mean utilities were found between pairs of health states differing in number of daily tablets (A vs. B) and frequency of injections (C vs. D) (Table 4). However, the utility difference between the two most preferred health states (A and D) was not significant. The majority of respondents (*n* = 152; 75.6%) had the same utility score for these two health states, while 23 participants (11.4%) had a higher utility for A, and 26 (12.9%) had a higher utility for D. There were no significant between-group differences in utilities by age, gender, or employment status.

**Table 4:** Comparisons between Health State Utilities^a^ (*N* = 201)

Comparison	Health States	Mean (SD) Health State Utility	Mean (SD) Difference Score^b^	95% Confidence Intervals for the Difference Score	T value (paired)	*p*-value
A vs. B	A. One daily tablet	0.908 (0.120)	−0.003 (0.013)	−0.005 to –0.002	−3.8	< 0.001
	B. Two daily tablets	0.905 (0.123)				
A vs. C	A. One daily tablet	0.908 (0.120)	−0.008 (0.088)	−0.020 to 0.004	−1.3	0.21
	C. Injections every month	0.900 (0.116)				
A vs. D	A. One daily tablet	0.908 (0.120)	0.002 (0.085)	−0.010 to 0.013	0.3	0.79
	D. Injections once every two months	0.910 (0.113)				
B vs. C	B. Two daily tablets	0.905 (0.123)	−0.004 (0.088)	−0.017 to 0.008	−0.7	0.48
	C. Injections every month	0.900 (0.116)				
B vs. D	B. Two daily tablets	0.905 (0.123)	0.005 (0.085)	−0.007 to 0.017	0.8	0.40
	D. Injections once every two months	0.910 (0.113)				
C vs. D	C. Injections every month	0.900 (0.116)	0.009 (0.026)	0.006 to 0.013	5.2	< 0.001
	D. Injections once every two months	0.910 (0.113)				

^a^TTO scores are on a scale anchored with 0 representing dead and 1 representing full health

^b^Difference scores were calculated by subtracting the utility of the first health state from the utility of the second health state. For example, the difference score for A vs. B was calculated by subtracting the utility of health state A (0.908) from the utility of health state B (0.905)

*CI* confidence interval; *SD* standard deviation

Additional exploratory descriptive analyses were conducted so that utilities could be reported separately for those who preferred oral ART and those who preferred injectable ART as reported during the ranking task (Table 5; Results described in online supplementary material).

**Table 5:** Time Trade-Off Utilities within Subgroups Categorized According to Preference for Either Oral or Long-Acting Injectable ART^a^

Health States	Participants who preferred oral treatment^b^		Participants who preferred long-acting injectable treatment^b^		Between-group difference score	
	Mean (SD)	95% CI	Mean (SD)	95% CI	Mean (SD)	95% CI
Total Sample (N = 201)	*n* = 78		*n* = 123			
A. Single tablet	0.921 (0.111)	0.896–0.946	0.900 (0.124)	0.878–0.922	0.021 (0.119)	−0.013–0.055
B. Two tablets	0.916 (0.118)	0.889–0.943	0.898 (0.126)	0.875–0.920	0.018 (0.123)	−0.017–0.054
C. Injections every month	0.885 (0.139)	0.853–0.916	0.910 (0.099)	0.893–0.928	−0.026 (0.116)	−0.062–0.010
D. Injections every two months	0.894 (0.139)	0.863–0.925	0.920 (0.092)	0.904–0.936	−0.026 (0.113)	−0.061–0.009
Subgroup of participants who differentiated between health states in the TTO task^c^ (N = 73)	*n* = 33		*n* = 40			
A. Single tablet	0.933 (0.115)	0.892–0.974	0.849 (0.169)	0.795–0.903	0.085 (0.147)	0.018–0.151
B. Two tablets	0.922 (0.132)	0.875–0.969	0.841 (0.170)	0.786–0.895	0.081 (0.154)	0.009–0.154
C. Injections every month	0.848 (0.168)	0.788–0.907	0.880 (0.115)	0.843–0.917	−0.032 (0.141)	−0.101–0.037
D. Injections every two months	0.870 (0.172)	0.809–0.931	0.909 (0.102)	0.877–0.942	−0.040 (0.138)	−0.108–0.028

^a^TTO scores are on a scale anchored with 0 representing dead and 1 representing full health

^b^Participants were categorized as preferring either oral or long-acting injectable ART based on their responses in the introductory ranking task that preceded the TTO utility elicitation. Participants were categorized as preferring oral treatment if they ranked an oral ART health state (A or B) as most preferred. Participants were categorized as preferring long-acting injectable treatment if they ranked a long-acting injectable ART health state (C or D) as most preferred

^c^This subgroup of 73 participants had TTO utility scores reflecting differences in preference among the four health states (i.e., at least one health state had a utility that was different from the utility of another health state). Each of the other 128 participants did not differentiate among health states in the TTO task, which means they had the same utility for all four health states (e.g., one of these participants could have had a utility of 0.90 for all four health states, while another could have had a utility of 0.85 for all four health states)

*ART* antiretroviral treatment; *CI* confidence interval; *LAI* long-acting injectable; *SD* standard deviation; *TTO* time trade-off

## Discussion

In this sample of PLHIV, preferences for treatment process varied, with some preferring LAI ART and others preferring daily oral treatment. The most frequently preferred treatment regimen was the LAI ART administered every two months. This result adds to previously reported findings suggesting that oral medication is not always preferred over injectable treatment options [30]. Current findings suggest that the LAI dose frequency of every two months is a welcome treatment option for many PLHIV, while other respondents were resistant to injections. Overall, findings suggest that LAI treatment would be a good fit for many but not all PLHIV.

Participants’ quotations (Table 3) highlight the wide range of reasons for treatment process preferences. PLHIV who preferred daily oral administration often mentioned a fear of needles and the time required for clinic visits with LAI treatment. In contrast, those who preferred the LAI health states often perceived the injectable dosing schedule to be more convenient and discreet than daily oral dosing.

Mean utility differences between health states were relatively small, which is common for treatment process utilities [29, 30, 47, 48]. Still, small utility differences can have an impact on the outcome of an economic model. The utility difference scores in Table 4 can be incorporated into a CUA to ensure that patient preference for route of administration and dose frequency is represented in economic models. These utility difference scores can be used to adjust utility values for treatment groups that differ by treatment process. For example, in a model comparing an LAI treatment administered every two months to a two-tablet daily regimen, modelers could adjust the utility of the oral treatment downward by 0.005 (i.e., difference between B and D in Table 4). This adjustment for treatment process can be included in either a base case analysis or a sensitivity analysis examining additional factors contributing to the value of ART.

To model some scenarios involving subgroups of PLHIV, it may be useful to use utilities from Table 5. Although health state D (LAI every two months) was most commonly preferred, there were some patients who feared needles and would never consider switching from oral to injectable treatment. In real-world clinical settings, LAI ART would be targeted only toward the patients who are open to injections, while patients who feared needles would simply continue daily oral treatment. In economic modeling of the LAI treatment, it may be appropriate to use utilities that specifically represent preferences of the target population (i.e., participants who ranked an injectable health state as most preferred), rather than those who are not part of the relevant treatment population. For example, in a model comparing LAI ART to a single daily tablet regimen for patients who would be open to LAI treatment, the utility of daily oral treatment could be adjusted downward by 0.02 (i.e., difference between A and D in Table 5).

This study’s data collection methods may have implications for future TTO studies. For vignette-based TTO utility elicitation, face-to-face individual interviews are optimal for ensuring that respondents understand the health states and TTO task, while minimizing erroneous logical inconsistencies that often emerge from online TTO studies [49]. Because the current study was conducted during the COVID-19 pandemic, face-to-face interviews were conducted by videoconference as described in the Methods section. The TTO results, which followed logical patterns consistent with respondent preferences, suggest videoconferencing is a viable alternative for TTO interviews. However, videoconferencing may not be appropriate for all TTO studies. The current sample consisted of patients with insight into the health states, rather than general population respondents without relevant experience. In addition, there were only four health states, which were relatively brief and easy to understand. Because of the specific sample and health states, less explanation and querying of seemingly illogical responses were necessary, compared to some other vignette-based studies. It may not be feasible to apply this videoconferencing approach to a study with a more complex set of health states valued by a general population sample.

Methodological limitations should be considered when interpreting results. The limitations of vignette-based methods have previously been described [34]. With all vignette-based utility elicitations, the resulting utilities represent preferences for health state descriptions rather than real-world experience. In the current study, the respondents had experienced many aspects of the health states, including living with HIV and receiving oral ART. However, only one of the 201 participants reported personal experience with LAI ART. It is possible that preferences could be different after patients have an opportunity to try the LAI treatment.

Characteristics of the sample are also associated with limitations. Although some health technology assessment agencies prefer that utilities are based on general population values [50–52], general population respondents may not have insight into the impact of daily oral medication. For example, people without HIV may not know what it is like to live with the stigma and daily reminder of HIV, which are uniquely important issues to this population. Therefore, this study was conducted with a patient sample. The extent to which utilities from the current study may differ from general population values is not known.

Generalizability of these UK preferences to other countries is also unknown. Because the LAI treatments are administered by medical professionals at clinics, the convenience of receiving injections varies by geographic region, medical system, and the distance between a patient’s home and a clinic. Preferences and utility values may differ in locations where it is more or less difficult to access medical treatment.

This study has implications for both clinical practice and economic modeling. While some PLHIV will always prefer oral ART, current results indicate that many patients may prefer LAI ART. LAI treatment could eliminate some challenges interfering with treatment adherence, which could lead to improved health outcomes. In addition, utilities estimated in this study may be useful in economic modeling comparing oral vs. LAI ART. By including these treatment process utilities in CUAs, modelers can help ensure that preferences of PLHIV are considered as part of decision-making about healthcare resource allocation.

## Supplementary Information

Below is the link to the electronic supplementary material.

Supplementary file1 (DOCX 28 kb)

## Data Availability

Data are available from the principal investigator upon reasonable request.
