# Supplementary material for: Patient preferences and health state utilities associated with the treatment process of antiretroviral therapy for people living with HIV
Source: Qual Life Res. 2022 Dec 13;32(2):531–41. doi: 10.1007/s11136-022-03290-0 (PMC9746581; doi:10.1007/s11136-022-03290-0)
Supplement: Supplementary file 1 — Supplementary file1 (DOCX 28 kb) [file 11136_2022_3290_MOESM1_ESM.docx]

**SUPPLEMENTARY MATERIAL**

**Table of Contents**

[I. TEXT OF THE HEALTH STATE VIGNETTES 1](#_Toc116049260)

[II. BACKGROUND INFORMATION DOCUMENT 3](#_Toc116049261)

[III. JUSTIFICATION OF THE HEALTH STATE DEVELOPMENT PROCESS 4](#_Toc116049262)

[IV. SAMPLE SIZE 5](#_Toc116049263)

[V. EXPLORATORY SUBGROUP ANALYSES IN TABLE 5 5](#_Toc116049264)

[VI. SAMPLE CHARACTERISTICS 6](#_Toc116049265)

# TEXT OF THE HEALTH STATE VIGNETTES

## Health State A: One Daily Tablet

### Human Immunodeficiency Virus (HIV)

- You have the viral infection called **HIV**. Although there is currently **no cure** for this infection, current treatments are very effective.
- Without treatment, HIV would cause your immune system to **weaken**, which can result in **life-threatening infections** and a range of **symptoms** (for example: pneumonia, fatigue, unhealthy weight loss, swollen glands, fevers, chills, night sweats, cough).
- When you are tested for levels of HIV in your blood, you have an **“undetectable viral load.”** This means that the test finds minimal or no sign of HIV in your blood.
- Because of your treatment, you have **no symptoms** related to HIV.
- HIV **does not interfere** with your ability to work or perform daily activities.
- Without treatment, you could pass HIV to other people through sexual contact. However, **with an undetectable viral load, there is no risk of transmitting the virus** to other people through sexual contact.

### Daily Oral - 1 Tablet

- You take **one tablet every day**.
- Your doctor recommends that you take this tablet at the same time every day.
- When you travel, you need to take these tablets with you so that you do not skip a day of treatment.

## Health State B: Two Daily Tablets

### Human Immunodeficiency Virus (HIV)

- [Same as the first six bullet points in health state A]

### Daily Oral - 2 Tablets

- You take **two tablets every day**. You take both tablets at the same time.
- Your doctor recommends that you take these tablets at the same time every day.
- When you travel, you need to take these tablets with you so that you do not skip a day of treatment.

## Health State C: Injections Every Month

### Human Immunodeficiency Virus (HIV)

- [Same as the first six bullet points in health state A]

### Long-acting Injectable - Once Each Month

- You are treated with **two injections once each month**.
- You receive both injections during one appointment **at a clinic or hospital**.
- It is important that you get these injections around the same day each month (within 7 days before or after the planned injection date).
- You are injected in the buttocks.
- The injections take only a **few minutes**.
- If you receive your injections as scheduled, you do **not** need to take any tablets for HIV.

## Health State D: Injections Once Every Two Months

### Human Immunodeficiency Virus (HIV)

- [Same as the first six bullet points in health state A]

### Long-Acting Injectable - Once Every Two Months

- You are treated with **two injections once every two months**.
- You receive both injections during one appointment **at a clinic or hospital**.
- It is important that you get these injections around the same day each month (within 7 days before or after the planned injection date).
- You are injected in the buttocks.
- The injections take only a **few minutes**.
- If you receive your injections as scheduled, you do **not** need to take any tablets for HIV.

# BACKGROUND INFORMATION DOCUMENT

*This material was presented to each participant before presenting the health states.*

**Treatment Options**

- Today, we will be discussing treatments for HIV. Two are **daily oral** treatments and the other two are **long-acting injectable** treatments.
- The treatments are equally effective, and they have similar rates of side effects.
- Research has shown that some patients prefer the **daily oral** treatment, while others prefer the **long-acting injectable** treatment.

**Daily Oral Treatments**

- **Some people living with HIV have reported things they like about the daily oral treatments, including the following:**
- Some people have said that taking oral tablets is easy.
- Some people have said that treatments requiring only one or two tablets taken once per day are easy.
- Some people say daily tablets are easy to fit into their daily routine.
- **Some people living with HIV have reported things they do not like about the daily oral treatments, including the following:**
- Some people worry about forgetting to take their tablets every day, which could have implications for their health and ability to transmit the disease to others.
- Some people have said their tablets serve as a daily reminder of their HIV.
- Some people worry about someone seeing their daily tablets and finding out about their HIV diagnosis.

**Long-Acting Injectable Treatments**

- **Some people living with HIV have reported things they like about the long-acting Injectable treatments, including the following:**
- Some people say the long-acting treatments are convenient because they do not have to take medication every day, and when they will be away from home, they do not have to plan to bring the medication with them.
- There is no concern about missing or forgetting a daily dose.
- There is no risk of people finding the medication, which means there is less risk of anyone learning you have HIV.
- **Some people living with HIV have reported things they do not like about the long-acting Injectable treatments, including the following:**
- Some people find it inconvenient to schedule clinic visits for the injections.
- Some people may be concerned about the time required for the clinic visits.
- Some people report some soreness from injections.

# JUSTIFICATION OF THE HEALTH STATE DEVELOPMENT PROCESS

In vignette-based utility studies, the health state vignettes are drafted to represent a typical patient experience.^[[1]](#footnote-1)^ While patients can each report their own experience, these individual experiences are not necessarily representative of the typical experience across the broader patient population. Therefore, to ensure that health states represented typical patient experiences with the routes of administration, the health state text was developed based on published research with large samples of PLHIV, as well as clinician input. The published literature provided insight into the experiences of a wide range of patients, identifying the treatment process attributes that tend to be most important to this patient population. In addition, clinicians who had treated many PLHIV were able to provide useful input because they could describe the typical experience with medication across the broad range of patients they had treated.

# SAMPLE SIZE

This study was conducted with a sample size target of 200 participants. Since the primary results of a vignette-based utility study are descriptive (i.e., the mean utility values), there is not a key statistical comparison on which a sample size should be based. Therefore, a power analysis was not applicable for determining the target sample size of this study. Instead, the sample size target was determined based on review of recent TTO studies published in peer-reviewed journals. Sample sizes of these studies vary widely, but most tend to be in the range of approximately 100 to 200 participants. Therefore, a sample size of 200 was considered to be adequate for the purpose of this study.

# EXPLORATORY SUBGROUP ANALYSES IN TABLE 5

Exploratory descriptive analyses were conducted so that utilities could be reported separately for those who preferred oral ART and those who preferred injectable ART as indicated during the ranking task (Table 5). In the subgroup of respondents who preferred oral treatment (n = 78), health states A and B had greater utilities than C and D (all p <0.05). In the subgroup that preferred injectable treatment (n = 123), utilities for D (injections every two months) were greater than those for health states A (p <0.05) and B (p <0.01) describing oral treatment. The utility of health state C (injections every month) was numerically greater than the utility of A and B, but these differences were not statistically significant.

When focusing only on the smaller subset of participants who had utility scores differentiating among health states (i.e., the 73 respondents who did not have the same utility score for all four health states), results followed the same pattern, but with greater differences between health state utilities. In the subgroup of 33 participants who preferred oral treatment, all pairwise comparisons between health state utilities were statistically significant (p <0.05). In the subgroup of 40 participants with preference for an LAI treatment, the following pairwise comparisons were statistically significant (p <0.01): A vs. B, A vs. D, B vs. D, and C vs. D.

# SAMPLE CHARACTERISTICS

The current sample was predominantly male (83.1%). This was expected as more than two-thirds of people seen for HIV care in England were male in every year from 2013 to 2020 (i.e., the most recent year for which statistics are available).^[[2]](#footnote-2)^ Analyses found no significant gender differences in utility, so there is no indication that this gender imbalance had an impact on the study results.

Another sample characteristic that should be considered when interpreting results is the health status of the respondents. To be eligible for this study, participants were required to be virologically suppressed and currently treated with ART. However, it is possible that the benefits of LAI ART may be greater in terms of both utility and health outcomes for individuals whose HIV is not well controlled due to difficulty storing oral medications or maintaining adherence to oral treatment regimens (e.g., individuals who are homeless). Therefore, it is possible that results from this specific sample could underestimate benefits of the LAI route of administration.

1. Matza LS, Stewart KD, Lloyd AJ, Rowen D, & Brazier JE. (2021). Vignette-based utilities: Usefulness, limitations, and methodological recommendations. *Value Health*, 24(6), 812-821. [↑](#footnote-ref-1)
2. Gov.UK. Official Statistics - HIV: Annual Data Tables. (Last updated: 2 December 2021). Available from: <https://www.gov.uk/government/statistics/hiv-annual-data-tables>. Accessed: Jun 11, 2022. [↑](#footnote-ref-2)
